# Stenotophomonas Maltophilia As Not Just a Mere Colonozer: Two Cases of Urinary Tract Infection and Multidrug-Resistant Respiratory Infection

**DOI:** 10.7759/cureus.23541

**Published:** 2022-03-27

**Authors:** Zaryab Umar, Usman Ilyas, Salman Ashfaq, Rubal Bhangal, Mahmoud Nassar

**Affiliations:** 1 Internal Medicine, Icahn School of Medicine at Mount Sinai, Queens Hospital Center, New York, USA; 2 Internal Medicine, Allama Iqbal Medical College, Lahore, PAK

**Keywords:** cap, vap, pneumonia, uti, stenotophomonas maltophilia

## Abstract

Stenotrophomonas maltophilia, though commonly reported as an opportunistic respiratory pathogen, has been known to cause a wide variety of illnesses, including urinary tract infection, biliary sepsis, bacteremia, and osteomyelitis. Malignancy and immunocompromised states are the biggest risk factors associated with Stenotrophomonas maltophilia infection. Being an emerging nosocomial infection globally, the bacteria should no longer be considered as just a mere colonizer, and emphasis should be laid on understanding the mechanisms of resistance, modes of prevention, and treatment.

We present the case of an 89-year-old Haitian American male with a past medical history of prostate adenocarcinoma and urinary retention following transurethral resection of the prostate, requiring an indwelling urinary catheter who presented to the emergency department with poorly draining Foley, hematuria, and urinary retention associated with suprapubic pain. Laboratory investigations revealed elevated creatinine, and urine analysis was suggestive of infection. The patient was admitted for the treatment of complicated urinary tract infection and acute kidney injury in the setting of urinary retention. Urine culture and sensitivity results revealed Stenotrophomonas maltophilia sensitive to trimethoprim/sulfamethoxazole, to which the patient responded well. During the course of the patient's hospital stay, his kidney function gradually improved.

We also present the case of a 68-year-old female with a past medical history of chronic tracheostomy dependence who presented to the emergency department for worsening fatigue and copious secretions from tracheostomy. Chest X-ray was suggestive of consolidation/edema, and the patient got admitted under the impression of septic encephalopathy due to pneumonia in a patient with tracheostomy. The patient received appropriate antibiotic therapy, and her mental status improved. However, the patient late developed respiratory distress, tachycardia, and tachypnea with worsening right-sided infiltrates on chest X-ray. The patient was started on vancomycin and cefepime for possible aspiration pneumonia. Cefepime was later changed to meropenem. Sputum culture and sensitivity results grew Stenotrophomonas maltophilia sensitive to meropenem which was continued. The patient's clinical status, laboratory and imaging findings improved over the course of her hospital stay.

## Introduction

Stenotrophomonas maltophilia is a glucose non-fermenting gram-negative bacillus that is free-living, mobile, aerobic, and oxidase-positive [1,2]. Though an organism of low virulence, its capability to survive in humid conditions, ability to form biofilms, and the various mechanisms of antibiotic resistance have increased the pathogen's capabilities of causing infections, especially in hospital settings [2]. The increasing use of antibiotics, advances in cancer treatment, and the increased placement of catheters and other devices are risk factors contributing to the rise in Stenotrophomonas maltophilia infection [3,4]. Recognized commonly as a respiratory pathogen, the organism has been known to cause urinary tract infections, biliary sepsis, bacteremia, and osteomyelitis [5-8]. Herein, we describe a patient with a chronic indwelling catheter and a patient with tracheostomy for over two years developing Stenotrophomonas maltophilia urinary tract infection and pneumonia, respectively. This emphasizes that the organism is not just a mere colonizer and can impact many other organ systems besides the respiratory system. It is imperative to prevent and treat it appropriately. 

## Case presentation

Case 1

An 89-year-old Haitian American male with a past medical history of prostate adenocarcinoma on androgen deprivation therapy, bladder wall mass status post biopsy showing invasive high-grade urothelial carcinoma with extensive necrosis, urinary retention status post transurethral resection of the prostate and placement of an indwelling urinary catheter, right-sided hydronephrosis status post right percutaneous nephrostomy tube placement, hypothyroidism, hypertension, and prediabetes, presented to the emergency department with a poorly draining Foley and hematuria along with suprapubic pain. Additionally, the patient reported generalized weakness but denied fever, diarrhea, loss of consciousness, dizziness, nausea, or vomiting. Laboratory results obtained at the emergency department indicated a hemoglobin level of 6.9 g/dL, a creatinine level of 2.2 mg/dL, and a lactate level of 4.4 mmol/L (Table 1). Urine analysis revealed cloudy urine, trace blood, large leukocyte esterase >50 white blood cells (WBC), trace bacteria, and yeast. Urology was consulted, and bladder irrigation was performed without significant hematuria or blood clots.

**Table 1:** Pertinent labs at the time of presentation and during the patient's hospital stay BUN - blood urea nitrogen

Lab (reference range and units)	Day 1 of admission	Day 13 of admission	Day 19 of admission
Hemoglobin (14.0-18.0 g/dL)	6.9	7.7	8.0
BUN (6-23 mg/dL)	38	20	22
Creatinine (0.70-1.20 mg/dL)	2.23	1.55	1.31
Lactate (0.6-1.4 mmol/L)	4.4	-	-

The patient was admitted to the medicine floor under the impression of complicated urinary tract infection and acute kidney injury likely in the setting of urinary retention. The patient was started on intravenous ciprofloxacin pending the results of antibiotic sensitivity testing. He received three doses of 400 mg of ciprofloxacin intravenously. The patient was maintained on gentle hydration, and a serial basal metabolic panel was performed to evaluate improvement in renal function. The patient did not receive any blood transfusion during his stay at the hospital. The patient's urine culture and sensitivity tests revealed >100,000 colony-forming unit (CFU)/ml of Stenotrophomonas maltophilia sensitive to trimethoprim/sulfamethoxazole and ceftazidime (Table 2). Ciprofloxacin was discontinued, and the patient was prescribed trimethoprim/sulfa for a total of nine days and ceftazidime 6 grams over four days. During the course of the hospital stay, the patient's blood urea nitrogen and creatinine levels improved, and repeat urine cultures were negative.

**Table 2:** Culture and sensitivity results

	Stenotophomonas maltophilia minimum inhibitory concentration (MIC)	Susceptibility
Ceftazidime	4	Sensitive
Levofloxacin	>4	Resistant
Trimethoprim.Sulfa	2/38	Sensitive

Case 2

A 68-year-old female with a past medical history of beta-thalassemia trait, heart failure with reduced ejection fraction, coronary artery disease, diabetes mellitus, hypothyroidism, gastroesophageal reflux disease, hyperlipidemia, migraines, osteoporosis, and chronic tracheostomy dependency, presented from home to be evaluated for worsening fatigue and excessive secretions from the tracheostomy. The patient started having copious secretions warranting clearing out the tracheostomy multiple times and worsening fatigue to the point that the patient could not get out of bed.

**Figure 1:**
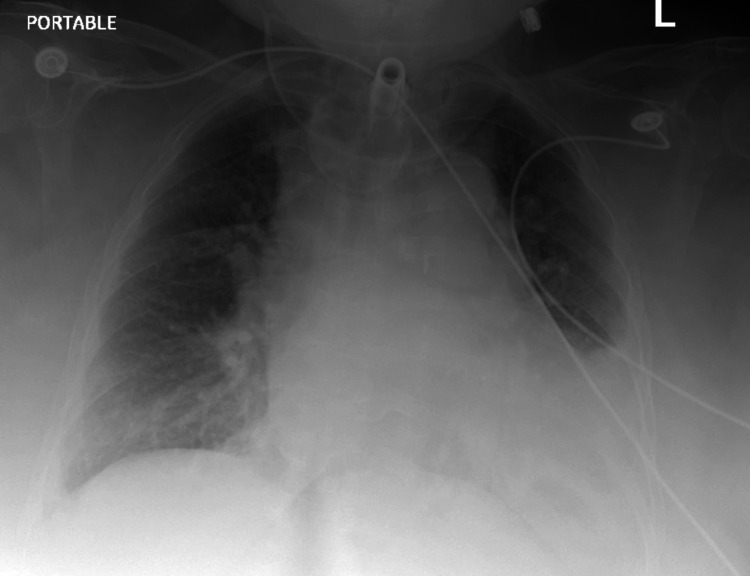
Chest X-ray at the time of admission revealing bilateral perihilar markings suggestive of pulmonary consolidation and/or edema Blunting of left mid and lower lung fields as well as the right costophrenic sulcus indicating pleural fluid.

According to the patient's husband, who was present at the bedside, the patient had undergone a tracheostomy two years ago while undergoing evaluation for gynecological malignancy, resulting in damage to the vocal cords. She and her husband have been performing tracheostomy care routinely for the past three years. At baseline, the patient could communicate and ambulate independently, but her ability to exercise decreased since the onset of her symptoms. Additionally, the patient had extensive edema. Upon inquiry, the husband informed that the patient had been drinking much water a few days before presenting to the emergency department. The patient had a white blood cell count of 12.69 upon presentation with chest X-ray findings of increased perihilar markings and blunting of the right costophrenic angle and left mid and lower lung fields (Table 3 and Figure1).

**Table 3:** Pertinent labs on the first day of admission and during the patient's hospital stay WBC - white blood cells; BUN - blood urea nitrogen

Lab (reference range and value)	Day 1 of admission	Day 11 of admission	Day 18 of admission	Day 23 of admission
WBC count (4.80-10.80 x10(3)/mcL)	12.69	8.04	18.65	8.99
BUN (6-23 mg/dL)	21	15	36	29
Creatinine (0.70-1.20 mg/dL)	0.87	1.53	2.24	1.08

The patient was admitted to the step-down unit with the impression of septic encephalopathy secondary to pneumonia in the setting of chronic tracheostomy. The patient was started on piperacillin/tazobactam for community-acquired pneumonia. Vancomycin was later added to increase coverage. The patient was also initially started on furosemide 40 mg every 12 hours in the setting of congestive heart failure. She was examined by point of care ultrasound at the bedside, which revealed a preserved left ventricular ejection fraction of 60% and a small, compressible inferior vena cava with some degree of intravascular depletion. The patient was started on continuous lactated ringer infusion after discontinuing furosemide and given a fluid bolus. In addition, she received albumin 5%. Her lower extremity edema continued but was likely in the setting of chronic venous stasis and the patient's body habitus and less likely due to congestive heart failure.

Additionally, her tracheostomy was noted to be leaking air. ENT was consulted to place a tracheostomy with a cuff to prevent air leakage. Cuff change was well tolerated, and the patient saturated well. Although the patient's mental status continued to improve, she continued to have diffuse secretions and required frequent suctioning.

**Figure 2:**
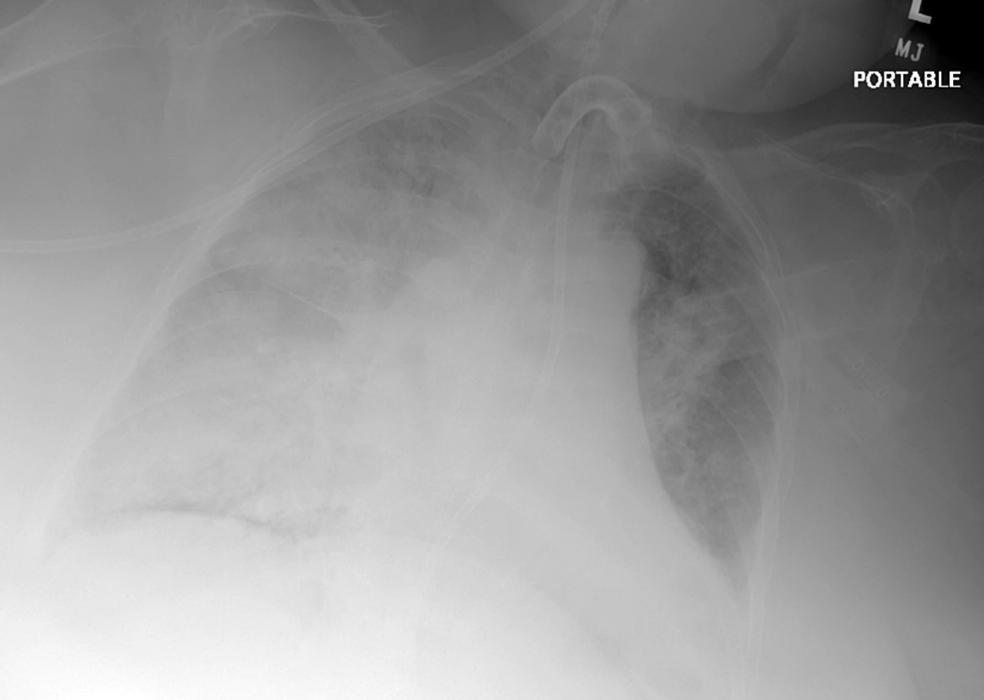
Chest X-ray obtained during the patient's episode of respiratory distress revealing increased/worsening right-sided pulmonary consolidation/edema

The patient was treated for pneumonia on medicine floors. Vancomycin was continued for seven days. Continuous positive airway pressure (CPAP) therapy was attempted daily to wean the patient off of mechanical ventilation, but the patient could not tolerate it. The patient was treated for generalized edema and pulmonary vascular congestion with furosemide 40 mg intravenously (IV) twice daily for seven days. Due to contraction alkalosis and improvement in fluid volume status, furosemide was reduced to 40 mg IV daily. The patient later developed significant respiratory distress, tachycardia, and tachypnea. The rapid response team was called. Chest X-ray revealed markedly worsened pulmonary edema on the right side (Figure 2). The laboratory results revealed an elevated D-dimer of 13,230 (normal at baseline) and an elevated troponin of 0.154 (Table 4). An electrocardiogram did not reveal any significant changes. DVT studies came back negative. Under the suspicion of pulmonary embolism as well as fluid overload, the patient was transferred to the step-down unit for more intensive management.

**Table 4:** Patient's troponin level at the onset respiratory distress and every six hours

Lab (reference range and value)	Day of the patient's respiratory distress, time (T)=0 hours	T=6 hours	T=12 hours	T=18 hours	T=24 hours	T=30 hours
Troponin (<=0.010 ng/mL)	0.154	0.471	0.460	0.336	0.343	0.256

In the step-down unit, the patient initially had a low blood pressure with a mean arterial pressure of 50 mmHg, a saturation of 97-98% on 100% fraction of inspired oxygen (FiO_2_), and positive end-expiratory pressure (PEEP) of five. Considering the patient's declining respiratory status and elevated D-dimers, therapeutic enoxaparin was prescribed to treat possible pulmonary embolism. Norepinephrine was administered for hypotension. A central line was placed. Additionally, cefepime 2 grams every 12 hours and vancomycin were prescribed for likely aspiration pneumonia. Troponin trended down, and there were no dynamic changes on EKG (Table 4). Given the hemodynamic instability, the patient was admitted to the intensive care unit for further management.

**Figure 3:**
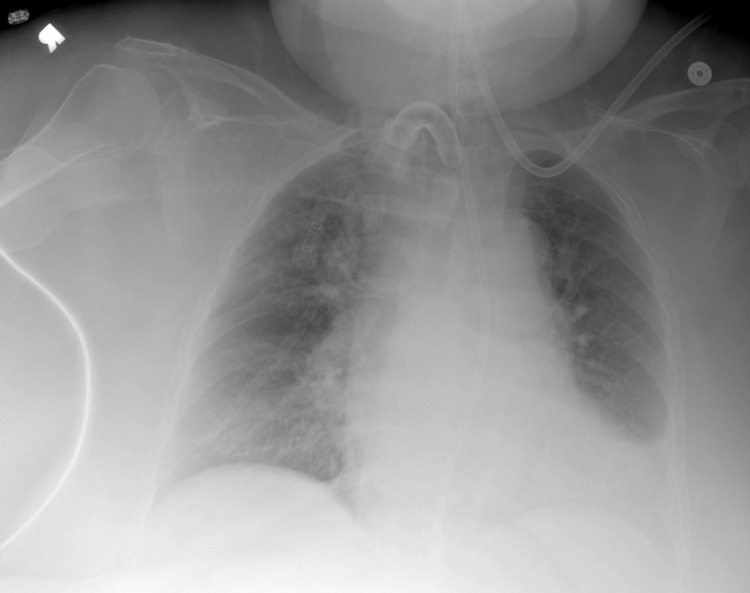
Chest X-ray obtained on the 23rd day of admission showing marked improvement of the right-sided pulmonary consolidation/edema

On admission to the ICU, the patient was placed on mechanical ventilation in volume control/assist control (VC/AC) mode, and FiO_2_ was titrated down from 100% to 50%. She continued to improve. The patient continued to receive albuterol inhalation and acetylcysteine nebulization. The patient was continued on cefepime and vancomycin by level. However, cefepime was later replaced by meropenem. The urine output was monitored and improved with IV furosemide. The chest X-ray was checked regularly and showed improvement in lung opacities (Figure 3). White blood cell counts trended down (Table 3). After an improvement in her condition, the patient was transferred to the step-down unit. Meropenem was continued for pneumonia. Blood culture results were negative. Sputum cultures were collected and grew multidrug-resistant Stenotrophomonas maltophilia and a few Pseudomonas, both sensitive to meropenem, which was given for a total of seven days (Table 5). CPAP trials were attempted; however, the rapid shallow breathing index remained above 100, so the trials were unsuccessful. Positive support ventilation (PSV) was also attempted; however, the patient required high levels of pressure support and was switched back to VC/AC mode. She was intermittently placed on synchronized intermittent mandatory ventilation (SIMV) along with the VC/AC mode, which she tolerated well.

**Table 5:** Culture and sensitivity results

	Stenotophomonas maltophilia minimum inhibitory concentration (MIC)	Pseudomonas aeruginosa minimum inhibitory concentration (MIC)	Susceptibility of Stenotophomonas maltophilia	Susceptibility of Pseudomonas aeruginosa
Amikacin		≤16		Sensitive
Aztreonam		8		Sensitive
Cefepime		4		Sensitive
Ceftazidime	4	4	Sensitive	Sensitive
Ciprofloxacin		0.25		Sensitive
Gentamicin		8		Intermediate
Imipenem		2		Sensitive
Meropenem	1	≤0.5	Sensitive	Sensitive
Piperacillin/tazobactam		≤1		Sensitive
Tobramycin		≤8		Sensitive
Trimethoprim/sulfa	0.5/9.5	≤2	Sensitive	Sensitive

## Discussion

Stenotrophomonas maltophilia, a gram-negative bacillus, is now emerging globally as an opportunistic, nosocomial infection with a high mortality rate. It is seen primarily in but not limited to immunosuppressed and debilitated patients [1,2,8]. The organism can be isolated from a wide variety of places such as the soil, water, animals, plants, and hospitals [1,2]. The table below mentions a few of the risk factors for Stenotrophomonas maltophilia infection among the general population (Table 6) [1,9]. 

**Table 6:** Risk factors associated with Stenotrophomonas maltophilia infection

#	Risk factors
1	Malignancy, particularly hematological malignancy
2	Human immunodeficiency virus (HIV)
3	Cystic fibrosis
4	Intravenous drug abuse
5	Surgical and accidental trauma
6	Prolonged hospitalization
7	Admission to ICU and mechanical ventilation
8	Indwelling vascular catheters and urinary catheters
9	Corticosteroids and immunosuppressive therapy
10	Prior treatment with broad-spectrum antibiotics
11	Gastrointestinal tract colonization and mucositis
12	Hematopoietic stem cell transplantation (HSCT)
13	Travel to hospital by air

The bacteria is predominantly reported as a respiratory pathogen but has also been reported to cause a wide variety of illnesses in immunocompromised populations, such as urinary tract infection, biliary sepsis, bacteremia, and osteomyelitis [5-7].

The ultramicrocells (UMC) of the bacteria are reported to be able to pass through 0.2-micrometer filters and can be isolated from water sources such as tap water and showers [10]. There are emerging concerns with the bacteria being a multi-drug-resistant organism (MDRO). It is believed that the bacteria acquire resistance in the community and retain the resistance when it comes in contact in clinical settings [10]. In the hospital setting, the bacteria is known to colonize and form biofilms on indwelling catheters that can act as a nidus for infections. 

Stenotrophomonas maltophilia is mostly reported to be resistant to third-generation cephalosporins, aminoglycosides, and antipseudomonal penicillins [8,10]. Resistance to beta-lactams is thought to be from the beta-lactamases, a zinc-containing penicillinase (L1), and a cephalosporinase (L2). Resistance to aminoglycosides is accounted for by the production of aminoglycoside transferase, and resistance to quinolones is thought to be because of the formation of efflux pumps [1,11].

Trimethoprim/sulfamethoxazole (TMP/SMX) remains the drug of choice in the treatment of Stenotrophomonas maltophilia; however, isolates have been reported that are resistant to this treatment [10]. The isolate found in our study was reported to be sensitive to TMP/SMX, and therefore treatment was continued with the medication. Phage therapy can be an effective way to prevent and treat the infection. Studies have suggested that cocktails of surfactant, antimicrobial peptides, and phage-coated catheters may provide alternative treatment and prevention for the organism; however, much more research is needed in this area in order to have conclusive evidence.

Emphasis should also be laid on disease prevention. Found naturally in portable water, patients can easily encounter the pathogen through the hospital tap water via touch, ingestion, inhalation, or at the hands of the health care workers [12,13]. The practice of adequate hand hygiene by medical professionals can play an important role in decreasing transmission to patients through tap water. Avoidance of hospital tap water for showering and cleaning wounds should be avoided as the bacteria can be easily transmitted through water, and patient education is key in avoiding this, especially amongst the population at risk. Avoidance of the formation of biofilms can also play a big role in preventing disease in vulnerable populations. Point of use water filters for faucets, showers, and ice machines that remove a wide variety of water-borne pathogens have been implemented in the hospital of the United States and is a cost-effective strategy to curb the rising cases of Stenotrophomonas maltophilia in the hospital setting [14].

## Conclusions

Stenotrophomonas maltophilia is an emerging nosocomial infection globally; it should no longer be considered as a mere colonizer while being reported. It is crucial to understand the risk factors associated with the bacterial infection, the possible mechanisms of resistance, and the appropriate management. In the first case, the patient had a history of malignancy and indwelling urinary catheter, while in the second case, the patient had a history of chronic tracheostomy dependence, which are considered to be risk factors associated with Stenotrophomonas maltophilia infection. In the first case, the patient's urine culture and sensitivity results grew the organism sensitive to trimethoprim/ sulfamethoxazole, and appropriate alteration of the antibiotic regimen resulted in improvement of the patient's clinical status. In the second case, the patient's sputum culture and sensitivity results grew the bacteria sensitive to meropenem which the patient was already receiving and hence continued. 
